# The Impact of Apolipoprotein E (*APOE*) Epigenetics on Aging and Sporadic Alzheimer’s Disease

**DOI:** 10.3390/biology12121529

**Published:** 2023-12-15

**Authors:** Madia Lozupone, Vittorio Dibello, Rodolfo Sardone, Fabio Castellana, Roberta Zupo, Luisa Lampignano, Ilaria Bortone, Antonio Daniele, Antonello Bellomo, Vincenzo Solfrizzi, Francesco Panza

**Affiliations:** 1 Department of Translational Biomedicine and Neuroscience (DiBrain), University of Bari Aldo Moro, 70121 Bari, Italy; ilariabortone@gmail.com; 2 Department of Orofacial Pain and Dysfunction, Academic Centre for Dentistry Amsterdam (ACTA), University of Amsterdam and Vrije Universiteit Amsterdam, 1081 HV Amsterdam, The Netherlands; vittoriodibello1@gmail.com; 3 Local Healthcare Authority of Taranto, 74121 Taranto, Italy; rodolfosardone@gmail.com; 4 Department of Interdisciplinary Medicine, Clinica Medica e Geriatria “Cesare Frugoni”, University of Bari Aldo Moro, 70121 Bari, Italy; castellanafabio@gmail.com (F.C.); zuporoberta@gmail.com (R.Z.); vincenzo.solfrizzi@uniba.it (V.S.); 5 Local Healthcare Authority of Bari, ASL Bari, 70132 Bari, Italy; luisalampignano@gmail.com; 6 Department of Neuroscience, Catholic University of Sacred Heart, 00168 Rome, Italy; antonio.daniele@policlinicogemelli.it; 7 Neurology Unit, IRCCS Fondazione Policlinico Universitario A. Gemelli, 00168 Rome, Italy; 8 Psychiatric Unit, Department of Clinical & Experimental Medicine, University of Foggia, 71122 Foggia, Italy; antonello.bellomo@unifg.it

**Keywords:** apolipoprotein E, Alzheimer’s disease, methylation, dementia, epigenetics, tau protein, amyloid-β, longevity

## Abstract

**Simple Summary:**

Modifications of gene expression (epigenetic alterations) have been implicated in the pathogenesis of complex diseases, such as Alzheimer’s disease (AD). Apolipoprotein E (ApoE), a major lipid carrier in the central nervous system, possesses three variants, E2, E3, and E4, with APOE4 increasing the risk of developing AD. The *APOE* gene undergoes epigenetic modifications. Diet, lifestyle, and pollutants might interact with the human genome. It is possible that environment and lifestyle can modify AD risk through epigenetic mechanisms involving the *APOE* gene and its promoter (a sequence of DNA to which proteins bind to initiate transcription) conditioning the imbalance between ApoE isoforms. One of the epigenetic mechanisms is DNA methylation at three sites of *APOE* CpG islands. A comprehensive interpretation of APOE-mediated effects within AD pathophysiology includes epigenetic mechanisms by which the equilibrium of its isoforms is regulated.

**Abstract:**

Sporadic Alzheimer’s disease (AD) derives from an interplay among environmental factors and genetic variants, while epigenetic modifications have been expected to affect the onset and progression of its complex etiopathology. Carriers of one copy of the apolipoprotein E gene (*APOE*) *ε4* allele have a 4-fold increased AD risk, while *APOE ε4/ε4*-carriers have a 12-fold increased risk of developing AD in comparison with the *APOE ε3*-carriers. The main longevity factor is the homozygous *APOE* ε3/ε3 genotype. In the present narrative review article, we summarized and described the role of *APOE* epigenetics in aging and AD pathophysiology. It is not fully understood how *APOE* variants may increase or decrease AD risk, but this gene may affect tau- and amyloid-mediated neurodegeneration directly or indirectly, also by affecting lipid metabolism and inflammation. For sporadic AD, epigenetic regulatory mechanisms may control and influence *APOE* expression in response to external insults. Diet, a major environmental factor, has been significantly associated with physical exercise, cognitive function, and the methylation level of several cytosine-phosphate-guanine (CpG) dinucleotide sites of *APOE*.

## 1. Introduction

The study of complex diseases is based on the association among epigenetics, gene variants, and environmental factors [1,2]. A mixture of many pathogenic pathways and gene expression networks determines the pathophysiology of Alzheimer’s disease (AD). The current model of AD is based on the amyloid-β (Aβ) hypothesis, in which a series of deterministic events may lead from tau protein and Aβ deposition to neurodegeneration and progressive decline of cognitive function. This conceptualization matches autosomal-dominant AD, defined as dominantly inherited AD with pathological confirmation, although it is less appropriate for sporadic AD. A probabilistic AD model connoted by three variants of the disease has been proposed: autosomal-dominant AD, apolipoprotein E (apolipoprotein E gene, *APOE*) ε4 allele-related sporadic AD, and *APOE* ε4 allele-unrelated sporadic AD [3]. These three variants suggested a reduced weight of the Aβ hypothesis, giving more importance to environmental factors and lower-risk genes [3].

Epigenetic modifications have been found in AD [4]. From a genetic point of view, the known risk loci (such as *ABCA7*, *APOE*, *CLU*, *CR1*, *BIN1*, *CD2AP*, *EPHA1*, *MS4A6A–MS4A4E*, and *PICALM* genes) [5], showed a low penetrance in causing AD, except for Aβ production-related genes, and none of them have been related to different AD pathogenic pathways. On the contrary, epigenetic alterations may modify transcriptional activity globally throughout different genes and multiple biological pathways. Epigenetic mechanisms may also explain the influence of environmental stimuli such as dietary patterns, harmful exposures, and lifestyle factors on phenotypic outcomes in individuals with the same genetic variants, also in AD [6]. Generally, the genetic sequence and epigenetic code are linked in a clear way according to the methylation mechanism. In fact, some single nucleotide polymorphisms (SNPs) are considered a common epigenetic mark because of the rearranging of cytosine-phosphate-guanine (CpG) dinucleotides with C nucleotide methylation. These CpG-altering SNPs may modulate DNA methylation levels in a *cis* or *trans* manner or they may modify gene transcription at regions enhanced of CpG known as CpG islands [7,8,9]. 

The *APOE* variants, respectively ε2 ε3, ε4, and ε^3^r, are determined by four haplotypes at the *APOE* locus (19q13.32), derived from the allele association of two common SNPs, rs429358 (*C*^3937^ → *T*) and rs7412 (*C*^4075^ → *T*), coding for the different protein isoforms [10]. These four *APOE* alleles are probably the most investigated variants in the human Caucasian genome [11]. Remarkably, the *APOE* exon 4 region, encompassing the ε2/ε3/ε4 allele variants, is a well-defined CpG islands-rich area. Moreover, the two common SNPs rs429358 and rs7412 are CpG-altering and modify the CpG content of this area. This *APOE* CpG island-rich area is a transcriptional enhancer with a specificity linked to the ε4 allele and cell-type [12].

Since the early 1990s, many reports have shown that *APOE* may play a fundamental role in AD neurodegeneration. For sporadic AD, *APOE* allele *ε*4 is a key genetic risk factor [13,14,15], with a semidominant inheritance [16], and associated to the ApoE4 isoform. The *APOE ε4* allele is a major AD risk factor in both men and women between 40 and 90 years, and for all ethnic groups. Conversely, in sporadic AD, the *APOE* allele *ε*2, associated to the ApoE2 isoform, could have a protective effect [17,18]. The risk for sporadic AD in *APOE ε*4-carriers is increased, but the presence of the *APOE ε*4 allele alone is not a causal factor for AD pathology [19]. In this context, epigenetics may represent a candidate for a point of overlapping among several genetic risk factors for AD, such as the *APOE* ε4 allele, and the AD pathophysiological processes. Human ApoE is a glycoprotein of 299-amino acids, traditionally binding phospholipids, and cholesterol. ApoE is produced in three common isoforms (ApoE2, ApoE3, and ApoE4) differing in two amino acid residues at positions 112 and 158, and one very uncommon isoform (ApoE3r) [12]. In AD pathophysiology, a global interpretation of *APOE*-mediated effects includes epigenetic mechanisms by which the homeostasis of its isoforms is regulated [20]. In the present review article, we briefly summarized and highlighted the complex epigenetic regulation of the *APOE* gene in aging and sporadic AD. 

## 2. The Role of Apolipoprotein E in Alzheimer’s Disease Pathogenesis

For sporadic AD, the *APOE 4* allele is the most important genetic risk factor, as well as for the earlier stages of cognitive decline represented by mild cognitive impairment (MCI) [21], but its expression is poorly understood. Astrocytes and activated microglia produced the major amount of ApoE in the brain. Having one *APOE ε4* allele conducts to a 4-fold increased risk of developing AD, while having two *APOE ε4* alleles conducts to a 12-fold increased risk, in comparison with the *APOE ε3*-carriers. Conversely, the uncommon heterozygous carriers of the *APOE ε2* allele have an AD risk 40% lower and the homozygous carriers have a further reduced risk [22]. In brain, *APOE ε4*-carriers with normal cognition displayed higher Aβ and tau burden than *APOE ε3*-carriers; conversely, *APOE ε2*-carriers had reduced global Aβ burden, without differences in regional tau burden or accumulation over time [23]. The contribution in AD pathogenesis from *APOE* involves not only Aβ aggregation and its clearance, but also tau-mediated neurodegeneration [24], microglia impairment [25,26], astrocyte reactivity [27], and blood–brain barrier disruption [28,29].

The three ApoE isoforms bind and transport Aβ peptides with differential affinity during AD pathogenesis [30,31], being highest for ApoE4, intermediate for ApoE3, and lowest for ApoE2 [32,33]. Therefore, their effects are also different concerning Aβ aggregation and clearance, but not Aβ production [34,35]. ApoE also can affect tau-mediated neurodegeneration and tauopathy by modulating microglial responses to Aβ plaque pathology [36,37,38]. Thus, different ApoE isoforms may be associated with an increased or reduced AD risk [31,32], based on different combined effects of ApoE isoforms on both deposits of Aβ and neurofibrillary tangles [39]. *APOE* and its ε2/ε3/ε4 alleles have been connected by several genetic studies to different disorders and physiological conditions. Epigenetic alterations could explain the association between *APOE* and its associated diseases, considering that disease-associated genetic signals may also reflect a site sequence architecture for epigenetic codes [12].

## 3. Apolipoprotein E, Human Longevity, and Alzheimer’s Disease

A genetic association of *APOE* with both human longevity and AD was found, but the mechanistic contribution of *APOE* in aging and long life is largely under investigation. *APOE* pleiotropic roles may be explained by its exceptional epigenetic properties. In the AD brain, these epigenetic changes could contribute to neural cell dysfunction. Additionally, DNA methylation modifications have been found on specific genes associated with AD pathology such as *APOE*. In the AD brain, it was shown that *APOE* CpG islands were differentially methylated in an *APOE*-genotype and tissue-specific way [40]. In the *APOE* CpG islands, AD typically showed a lower level of DNA methylation occurring in brain regions affected by AD pathophysiology (highest levels in the cerebellum, with moderate levels in the hippocampus and the lowest levels in the frontal lobe). However, in the *APOE* CpG islands, there was a complex interplay among the presence of the *APOE ε4* allele, AD status, and DNA methylation levels. AD-specific methylation differences were mainly attributed to the *APOE* ε3/ε4 heterozygous subjects [40]. Allele variations in the major *APOE* CpG islands of targeted replacement (TR) mice expressing human *APOE* may affect its methylation in the brain [41]. Epigenetic changes may link modified gene expression with environmental stimuli such as dietary patterns and physical exercise. In animal models, *APOE* alleles may have alterations in epigenetic regulation responding to external stimuli reported in studies on *APOE* TR mice [42]. 

In the strategy for replacing mouse ApoE in the *APOE* TR models, we should consider differences between human and mouse *APOE* gene clusters, the complexity of transcriptional control of human *APOE*, and the structure of the targeting construct [43]. Moreover, lifestyle factors like education, smoking, alcohol consumption, and physical activity may weaken genetic risk in the process of age-related cognitive decline and dementia. At this regard, twelve potentially modifiable risk factors have been investigated to prevent or delay up to 40% the risk for different types of dementias including AD, i.e., lower educational level, hypertension, hearing loss, smoking, obesity, depressive syndromes, physical inactivity, diabetes, low social contact, immoderate alcohol consumption, air pollution, and traumatic brain injury [44]. The complex interactions among lifestyle, genetics, and age-related cognitive decline may encourage behaviors maintaining cognitive health in older age, including dietary habits [45]. At this regard, ApoE may be important for the pathophysiology of lipid metabolism [46] and central nervous system (CNS), although the role in healthy aging and longevity has seen its value grow [47,48,49]. 

In the lipid metabolism pathophysiology, ApoE may be related with normal/pathological aging, while its function in CNS pathophysiology needs further clarification [50]. In fact, in the CNS, there was about a quarter of total body cholesterol that may exert a significant impact on synaptic plasticity [51]. With advancing age, cholesterol metabolism may modify, and its related brain changes may be associated with the pathophysiology of AD [51]. So, in longevity and healthy aging, lipid and cholesterol maintenance are a critical factor also from an interventional point of view. Dietary interventions may manage the detrimental effects of the *APOE ε*4 allele [52], with a Mediterranean dietary pattern potentially including higher n-3 polyunsaturated fatty acid (PUFA) intakes [53,54]. 

Studies on longevity and healthy aging are related because subjects who live longer tend to be healthier for a greater part of their lives [55]. Healthy aging can be described as achieving older age maintaining intact cognition and/or mobility and without disabilities or multimorbidity. This last can be defined as the coexistence of two or more chronic diseases in the same subjects [56]. The detrimental effects of the *APOE* ε4 allele on longevity could influence the probability of a long human lifespan [55]. The *APOE* ε2 allele has a greater frequency in long-lived individuals than the ε4 allele [57]. Thus, the main longevity factor is the *APOE* ε3/ε3 genotype. The greater frequency of the ε3 allele in older individuals and their offspring than in controls derives from the higher amount of the homozygous *APOE* ε3/ε3 genotype in comparison with the ε2/ε3 or ε3/ε4 genotypes [58]. 

## 4. Specific Epigenetic Modifications of Apolipoprotein E in Alzheimer’s Disease

**Figure 1:**
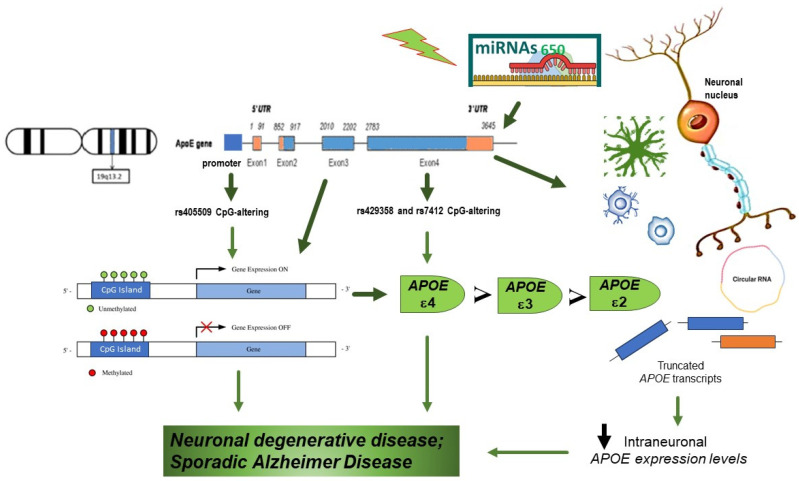
Apolipoprotein E *(APOE)* gene pleiotropic roles may be explained by its exceptional epigenetic properties. The APOE ε2/ε3/ε4 alleles are produced by two cytosine-phosphate-guanine (CpG)-altering SNPs (rs429358 and rs7412) in the core region of the APOE CpG islands. *APOE* ε4 carriers have the greatest number of CpG dinucleotide sites, while *APOE* ε2 carriers have the smallest number, so methylation levels for most CpG sites are in the order of *APOE* ε4 carriers > *APOE* ε3/ε3 > *APOE* ε2 carriers. In the promoter region, there is another SNP, rs405509. Furthermore, other epigenetics mechanisms are linked to the processing of primary miR-650 to mature miR-650 is mis regulated. miR-650 can significantly reduce the expression of *APOE*.

In response to environmental stimuli, epigenetic marks and signals may enable temporal combination of regulatory events through mechanisms including DNA methylation, histone modification/chromatin conformation, and noncoding microRNAs (miRNAs). In the *APOE* gene, several studies investigating DNA methylation suggested an age-dependent flow and *APOE* DNA methylation specific for brain area. The impact of aging on *APOE* methylation is based on the general link between DNA methylation and longevity, although current studies on the association among aging and *APOE* methylation patterns are limited and with little sample size as later described. The *APOE* genomic sequence is approximately 4 kb in size (chromosome19:45408714-45412650, hg19) including its promoter. This region encompasses 172 CpG dinucleotides [59]. At the *APOE* locus, three functional SNPs may modify DNA methylation, and of these, rs405509 is in the promoter region, while the other two SNPs (rs429358 and rs7412), which define the *APOE* e2/e3/e4 isoforms, are within exon 4 (Figure 1). Genetic variability in the *APOE* neural expression may contribute to the risk of AD considering that relative *APOE* ε4 mRNA expression is higher in AD patient than in healthy controls [60]. Moreover, together with the qualitative effect on the AD risk of the *APOE* e2/e3/e4 polymorphisms, functional *APOE* promoter mutations may determine quantitative variation of expression of these alleles that is a fundamental determinant of AD occurrence. In the late 1990s and early 2000s, polymorphic sites in the first intron and the proximal promoter the of *APOE* gene cluster (−1019 to +407) affecting *APOE* expression were identified, showing the deleterious effect of the Th1/E47cs T allele and the protective effect of the −491 T allele [61,62,63,64,65] (Table 1). Notably, these polymorphisms have been related with a differential AD risk [66,67,68]. 

In AD, the association between these polymorphic sites and the variability of sequence in the proximal promoter with ApoE protein levels are not clearly understood. In fact, among different studies, findings on the levels of expression of *APOE* RNA and the relationship with the ApoE levels varied. In human AD postmortem brain, there was elevated methylation in frontal lobe of a 5′-C-phosphate-G-3′ (CpG) island overlapping with exon four and downstream [69]. *APOE* has a well-defined CpG island external to the promoter region and overlapping with the *APOE* 3′-exon. In the human genome, these 3′-CpG islands are very rare, representing < 1% of the total CpG islands, and are also conserved in other mammals [70,71]. However, the *APOE* CpG island methylation level relates to the level of expression of four known *APOE* transcripts. The majority of the total *APOE* mRNA, with higher expression in the AD frontal lobe than in the frontal lobe of control subjects, is constituted by circular RNAs, mRNAs, and truncated *APOE* transcripts. The findings of several studies suggested several changes in the epigenome and the regulatory role of epigenomic elements related to the risk or clinical presentation of several neurological diseases, although the exact clinical significance of these signatures in the quantities of RNA and methylation level of CGI in the *APOE* 3′-exon was still unclear [69] (Table 1).

At the level of the individual CpG site, epigenetic regulation was shown by up/down patterns in the methylation profiles between samples and tissues. Significant differences in the global methylation levels among several regions of the brain were discovered across postmortem brain tissues. In brain regions primary affected by AD such as frontal lobe, temporal lobe, and hippocampus, methylation levels were lower. Conversely, we observed in the cerebellum, a region apparently without profound pathological alterations in AD but with recent important findings, the highest methylation levels, suggesting a correlation between the methylation levels of the *APOE* CpG islands and the vulnerability of brain regions in AD patients [40]. In fact, age- and AD-related alterations in several cerebellar subregions may also impact numerous functional domains, especially those affecting cognitive processing [72].

Genetic variants, which consist of CpG-altering SNP, can modify DNA methylation levels. These genetic variations may act like regulatory elements connecting genetic changes not only with the protein isoforms, but also with epigenetic variability [73] (Table 1). As previously described, the *APOE* ε2/ε3/ε4 alleles are produced by two CpG-altering SNPs (rs429358 and rs7412) residing in the core region of the *APOE* CpG islands. The *APOE* ε4 allele, if compared with ε2 or ε3 alleles, adds one more CpG, further saturating a small 12 bp region with 4 CpG sites. On the contrary, the *APOE* ε2 allele eliminates 1 CpG and opens a 33-bp CpG-free region. Consequently, these two SNPs may alter the regional CpG burden and probably influence global DNA methylation of the CpG islands (Figure 1). These CpG load changes might change the binding profiles of methyl-CpG binding domain proteins, associated to methylated DNA through their exclusive amino acid patterns [74]. 

**Figure 2:**
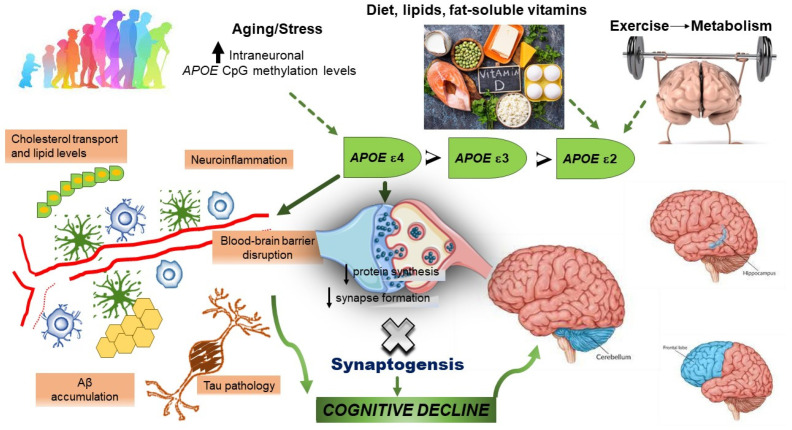
Despite the high lifetime risk linked to the presence of the *APOE* ε3/ε4 and *APOE* ε4/ε4 genotypes (the greatest risk factor for developing Alzheimer’s disease, AD), stochastic factors (such as environment, diet, physical exercise, and aging), may play a significant role as epigenetic modifiers, influencing the imbalance among the different ApoE isoforms. The role of *APOE ε4* allele in AD pathogenesis involves not only amyloid-β aggregation and clearance, but also tau-mediated neurodegeneration, microglia dysfunction, astrocyte reactivity, and blood–brain barrier disruption. These changes may only occur in brain regions profoundly affected by AD pathophysiology (highest levels in the cerebellum, with moderate levels in the hippocampus and the lowest levels in the frontal lobe).

Furthermore, within the *APOE* CpG islands, there is evidence of indirect indicators of protein binding which consist of histone marks and a DNase I hypersensitivity cluster. These findings suggested that the *APOE* CpG islands and exon 4 may be a site for chromatin remodeling and protein binding. Considering that environmental stimuli could influence DNA methylation gradually with aging, the differences in *APOE* CpG island methylation between healthy subjects and patients with AD increased with age [75]. Taken together, different methylation scenarios may be represented by the inheritance of different ε2/ε3/ε4 alleles in the *APOE* CpG islands, which could accumulate or change continuously with age, also modified by environmental factors. Recent results showed that methylation levels for most CpG sites may be in the order of *APOE* ε4-carriers (greatest number of CpG sites) > *APOE* ε3/ε3-carriers > *APOE* ε2-carriers (smallest number of CpG sites) [76] (Table 1). These changes could potentially alter protein binding, with some consequences on biological systems, even affecting the pathophysiological processes of multiple diseases and plasma lipids levels. *APOE* methylation could partially mediate the effects of age on plasma lipid (Figure 2).

**Table 1:** Overview of studies illustrating epigenetic signatures of apolipoprotein E gene (*APOE*) in aging and Alzheimer’s disease (AD).

*APOE* Exons, Promoter, and CGI				
Study	Study Design	Sample Size	Age or Mean Age at Death (Years)	Principal Findings
Foraker et al., 2015 [40]	Cross-sectional	AD: 15 Controls: 10	AD: 82.7 ± 9.3 Controls: 84.9 ± 8.9	In the cerebellum there was the highest levels of methylation (marginal mean = 93%), with lower levels in the hippocampus (marginal mean = 85%), and the lowest levels in the frontal lobe (marginal mean = 77%) of AD brain compared to controls. There was a complex interaction among the presence of the *APOE* ε4 allele, AD status, and DNA methylation levels in the *APOE* CpG islands. AD-specific methylation differences were mainly attributed to the ε3/ε4 heterozygous subjects
Lambert et al., 1997 [60]	Cross-sectional	Frontal lobe ε3ε4, ε2ε4, ε2ε3 AD cases: 14 Controls: 12	AD: 74.1 ± 11.8 (five male and nine female) controls: 83.0 ± 10.6	In heterozygotes AD, *APOE* ε4 mRNA expression is increased in patients with AD compared with healthy controls: genetic variability in the neural expression at the *APOE* locus contributes to AD risk. *APOE* ε3ε4 heterozygote subjects (high ε4 expressors and/or low ε3 expressors) were more likely to develop AD than subjects with high ε3 expressors and/or low ε4 expressors
Lee et al., 2020 [69]	Cross-sectional	Frontal lobe AD: 44 Controls: 21 Cerebellum AD: 51 Controls: 25	Frontal lobe AD: 86.8 ± 6.9 Controls: 87.9 ± 8.6 Cerebellum AD: 74.6 ± 9.3 Controls: 73.5 ± 10.9	*APOE* has a single CpG island overlapping with its 3′-exon. *APOE* circular RNA and full-length mRNA each constitute one third of the total *APOE* RNA, with truncated mRNAs likely constituting some of the missing fraction. All *APOE* RNA species had significantly higher expression in AD frontal lobe than in controls, suggesting a possible modified mechanism of gene action for *APOE* in AD involving also an epigenetically regulated transcriptional program driven by DNA methylation in the *APOE* CpG island
Yu et al., 2013 [73]	Cross-sectional	Frontal lobe AD: 9 Controls: 6	AD: 86.8 ± 6.9 Controls: 87.9 ± 8.6	*APOE* 3′-exon CpG island exhibited transcriptional enhancer/silencer activity, modulating expression of genes at the *APOE* locus in a cell type-, DNA methylation- and ε2/ε3/ε4 allele-specific manner. These results suggested a novel functional role for a 3′-exon CpG island involving the protein isoforms and also an epigenetically regulated transcriptional program
Ma et al., 2015 [76]	Cross-sectional	475 men and 518 women	18–87	The 13 *APOE* CpG sites were categorized into three groups: Group 1 exhibited hypermethylation (>50%, in the promoter region), Group 2 showed hypomethylation (<50%, in the first two exons and introns), and Group 3 exhibited hypermethylation (>50%, in the exon 4). *APOE* methylation was significantly associated to age and plasma total cholesterol. *APOE* methylation patterns differed across *APOE* ε variants and the promoter variant rs405509, which further had a significant interaction with age
Wang et al., 2008 [77]	Cross-sectional	Prefrontal cortex AD: 24 Matched controls: 10 Blood samples AD: 6 Matched controls: 6	AD: 80.9 ± 9.3 matched controls: 80.0 ± 9.8 Blood samples AD: 81 ± 4.5 matched controls: 80.0 ± 5.2	In the AD brain samples, a notably age-specific epigenetic drift was identified, suggesting a role of epigenetic effects in the AD development. *APOE* gene is of bimodal structure, with a hypomethylated CpG-poor promoter and a fully methylated 39-CpG-island, containing the sequences for the ε4-haplotype

CpG: 5′-C-phosphate-G-3′; CpG: cytosine-phosphate-guanine.

In the epigenetic scenario, miRNAs are known to be small non-coding RNAs with a length of ~22 nucleotides. They are also implicated in AD, as shown by the altered expression of miRNA 650 (miR-650) in AD brains [78]. Bioinformatic analysis showed that miR-650 may target the expression of three AD-related components: *APOE*, presenilin 1 (PSEN1), and cyclin-dependent kinase 5 (CDK5), with recent findings confirming that miR-650 may reduce in vitro the expression of *APOE*, PSEN1, and CDK5 [78].

## 5. Epigenetics of Apolipoprotein E and Cognitive Function: Contrasting Evidence in Alzheimer’s Disease

Several lifestyle and environmental stimuli could explain the effects of *APOE* genotype on AD and cognitive functioning, such as exercise [79], education [80], and vitamin D status [81] (Figure 2). Vitamin D is often referred to as a neurosteroid with neuroprotective and anti-inflammatory properties. Although the interplay between *APOE* genotype and vitamin D metabolism or transport in the nervous system is yet to be established, it is known that *APOE* contributes to the transport of lipid-soluble vitamins in the circulation and influences several immunological, inflammatory, and neurodegenerative processes [82]. Furthermore, *APOE* polymorphism and dietary responsiveness to fat-soluble vitamins, flavonoids, and n-3 PUFA were described, indicating *APOE* ε3 as a more flexible and responsive genotype than *APOE* ε4 [82]. Among implications for the development and progression of AD, vitamin D supplementation may be another potential strategy to consider for the *APOE* ε4 allele-carriers. Some reports showed that higher vitamin D concentrations in *APOE* ε4 homozygous carriers allow them to perform better at memory scores [83]. Then, compared to the *APOE* ε3/ε3-carriers, the *APOE* ε4-carriers showed earlier onset of cognitive impairment in AD. However, after the disease onset, the effect of the *APOE* genotype on the progression of cognitive impairment remained debated [84]. 

For this reason, epigenetic modifications of *APOE*, such as DNA methylation, may have a key role in maintaining intact cognitive function in older age. Growing DNA methylation levels at the *APOE* promoter region were found on postmortem prefrontal cortex samples of sporadic AD individuals using MALDI-TOF mass spectrometry and lymphocytes [77]. A notably age-specific epigenetic drift was identified, supporting a potential role of epigenetic effects in AD development. These results may partly explain the differences between *APOE* ε4 carriers and noncarriers in the benefits triggered by long-term exercise that might depend, at least partially, on mechanisms of metabolic response of prefrontal cortex to physical activity [79].

Numerous studies have indagated the relationship between *APOE* DNA methylation and AD or MCI [85,86,87]. Instead, the association between *APOE* DNA methylation and cognitive function in healthy subjects without cognitive impairment was evaluated by two studies, with controversial findings [88,89]. Liu and colleagues found an inverse association between DNA methylation in the *APOE* gene region and delayed recall capacity among 289 older African American people with a mean age of 67 years during normal cognitive aging [88]. Conversely, the other study, conducted in a large European cohort, observed no association between general cognitive functioning and *APOE* DNA methylation [89].

Many reports have suggested that neuroinflammation may have a key role in AD pathogenesis [90]. Dietary habits are known to influence systemic inflammation, neuroinflammation, and inflammaging [91]. A recent study conducted in a cohort of racially diverse middle-aged people (*n* = 411), pursued to identify DNA methylation sites associated with cognitive function in the genomic region of *APOE*. Regarding the inflammatory potential of the diet, among the dietary inflammatory index, cognitive performance, and the methylation level of several CpG sites have been detected significant relationships [92]. 

However, studies are contrasting in this regard, and whether epigenetic biomarkers could be used for predicting AD is still unclear. In the *APOE* gene, DNA methylation at two CpG sites (3/13) that are known to show age-dependent changes was related with the total cholesterol and high-density lipoprotein cholesterol ratio, but not with cognitive status, family history of AD, or the risk of cardiovascular disease in a blood-based DNA methylation study of 5828 people from the Generation Scotland cohort [89]. These findings supported that there is no evidence yet for considering *APOE* methylation as a biomarker for predicting AD or cardiovascular disease, although *APOE* methylation was associated with the blood levels of cholesterol [89].

Some limitations could affect specific methodologies used in the studies cited for assessing DNA methylation of *APOE*. Overall, at the transcriptional level, all major cell types have AD pathology, and single cell-level resolution may be critical; moreover, changes in gene expression, including directionality, can be conditional on cell type. The number of significant differentially expressed genes for non-neuronal populations were substantially smaller, likely reflecting reduced power in lower-abundance cell types [93]. Given that astrocytes are the primary producers of brain ApoE, alterations of epigenetically regulated *APOE* expression in glia may explain a significant part of the genetic AD risk linked to this gene [94]. Furthermore, although several imputation methods exist, a major deficiency lies in the inability to cope with large datasets, such as DNA methylation chips. Therefore, specific methods for imputing missing methylation data are needed [95].

## 6. Conclusions

In the panorama of current available evidence, the investigation of healthy aging and longevity is currently of remarkable interest. *APOE* could be considered an epigenetic mediator of senescence considering that different ApoE biochemical pathways in lipid metabolism, neuroinflammation, and neurodegeneration may contribute to longevity and healthy aging. Nonetheless, such areas of investigation are still increasing, since ApoE function in neurodegenerative diseases, particularly AD, cannot be uniquely explained by ApoE effects in lipid metabolism. Furthermore, the imbalance in the ApoE isoforms could explain the pathophysiological process of cognitive impairment linked to sporadic AD [20]. 

Stochastic factors (such environmental, diet, and pollution) may play a significant role in sporadic AD, despite the elevated lifetime risk linked to *APOE* ε3/ε4 and *APOE* ε4/ε4 genotypes. Indeed, according to the notion of stochastic risk or protective factors and although it is known that *APOE* ε4/ε4-carriers developed dementia about 10 years earlier than *APOE* ε2 carriers [96], there was still significant discrepancy in the age of onset for *APOE* ε4/ε4-carriers [22]. During the process of aging, the accumulation of molecular changes driven by genetic and epigenetic events in the organism lead to a loss of phenotypic plasticity over time. Also, epigenetics may be altered during aging processes, and this is particularly important, as age is the greatest risk factor for developing AD [97]. 

The epigenetic hallmarks of *APOE* and the impact of *APOE* epigenetics on aging and sporadic AD are mainly obtained from cross-sectional studies, while additional longitudinal findings are needed to obtain biomarkers of DNA methylation helpful for directionality of gene expression. Future longitudinal studies with methylation profiles are needed to provide a link between the *APOE* epigenetics and aging-related phenotypes and neurodegenerative disorders, including possible treatment. There is only one agent, the gene therapy LEX 1001, in the pipeline targeting the *APOE* ε4 allele. In this trial, a viral vector carrying the *APOE* ε2 allele is being given to antagonize the effects of *APOE* ε4 [20]. Some categories, i.e., epigenetic agents, have few therapeutic approaches in the pipeline; the present understanding of the biology of these processes may not have matured sufficiently to suggest target mechanisms for disease-modifying therapies [98]. Recent progress in the understanding of AD pathogenesis suggested that dysregulation of mRNA biogenesis may be involved in neurodegeneration. New methodological advances of AD treatment have developed short and synthetic antisense oligonucleotides that recognize target mRNA for posttranscriptional regulation to correct protein expression errors [99]. Dietary interventions may represent the most effective strategies in managing and regulating the onset of age-related diseases in humans, although they have not provided the promotion of longevity [100].

## Data Availability

The datasets analyzed during the current review are available from the corresponding authors (M.L. and F.P.) upon reasonable request.
